# The Role of MSC Therapy in Attenuating the Damaging Effects of the Cytokine Storm Induced by COVID-19 on the Heart and Cardiovascular System

**DOI:** 10.3389/fcvm.2020.602183

**Published:** 2020-12-09

**Authors:** Georgina M. Ellison-Hughes, Liam Colley, Katie A. O'Brien, Kirsty A. Roberts, Thomas A. Agbaedeng, Mark D. Ross

**Affiliations:** ^1^Faculty of Life Sciences & Medicine, Centre for Human and Applied Physiological Sciences, School of Basic and Medical Biosciences, King's College London Guy's Campus, London, United Kingdom; ^2^School of Sport, Health, and Exercise Sciences, Bangor University, Bangor, United Kingdom; ^3^Department of Physiology, Development, and Neuroscience, University of Cambridge, Cambridge, United Kingdom; ^4^Research Institute for Sport and Exercise Sciences, Liverpool John Moores University, Liverpool, United Kingdom; ^5^Faculty of Health & Medical Sciences, Centre for Heart Rhythm Disorders, School of Medicine, The University of Adelaide, Adelaide, SA, Australia; ^6^School of Applied Sciences, Edinburgh Napier University, Edinburgh, United Kingdom

**Keywords:** COVID-19, mesenchymal stem cells, cytokine storm, cardiovascular, regeneration and repair

## Abstract

The global pandemic of severe acute respiratory syndrome coronavirus 2 (SARS-CoV-2) that causes coronavirus disease 2019 (COVID-19) has led to 47 m infected cases and 1. 2 m (2.6%) deaths. A hallmark of more severe cases of SARS-CoV-2 in patients with acute respiratory distress syndrome (ARDS) appears to be a virally-induced over-activation or unregulated response of the immune system, termed a “cytokine storm,” featuring elevated levels of pro-inflammatory cytokines such as IL-2, IL-6, IL-7, IL-22, CXCL10, and TNFα. Whilst the lungs are the primary site of infection for SARS-CoV-2, in more severe cases its effects can be detected in multiple organ systems. Indeed, many COVID-19 positive patients develop cardiovascular complications, such as myocardial injury, myocarditis, cardiac arrhythmia, and thromboembolism, which are associated with higher mortality. Drug and cell therapies targeting immunosuppression have been suggested to help combat the cytokine storm. In particular, mesenchymal stromal cells (MSCs), owing to their powerful immunomodulatory ability, have shown promise in early clinical studies to avoid, prevent or attenuate the cytokine storm. In this review, we will discuss the mechanistic underpinnings of the cytokine storm on the cardiovascular system, and how MSCs potentially attenuate the damage caused by the cytokine storm induced by COVID-19. We will also address how MSC transplantation could alleviate the long-term complications seen in some COVID-19 patients, such as improving tissue repair and regeneration.

## Introduction

As of 3rd November 2020, there are >47 million cases of the coronavirus 19 or severe acute respiratory syndrome coronavirus 2 (SARS-CoV-2) that causes coronavirus disease 2019 (COVID-19) in the World. There have been >1.2 million reported deaths due to COVID-19, and >34 million infected cases have recovered. As it stands, the infection and death rate due to COVID-19 is below that of previous pandemics. For example, the 1918 Spanish flu outbreak saw 500 million people infected throughout the World and 17–50 million people died over a 2 year span; with up to 25 million deaths in the first 25 weeks (1). Prior to the 1918 flu pandemic, influenza outbreaks had only killed juveniles and the elderly or already weakened patients. However, the Spanish flu was killing completely healthy young adults, while leaving children and those with weaker immune systems still alive (2). This high mortality was attributed to malnourishment, overcrowded medical camps and hospitals, and poor hygiene, all exacerbated by the recent war which promoted bacterial superinfection (3). The outcome of the COVID-19 pandemic is impossible to predict, however history shows that past pandemics have reshaped societies in profound ways. It is clear that COVID-19 has already changed the World and the way we live and work forever.

SARS-CoV-2 gains entry to human cells through the angiotensin-converting enzyme 2, or ACE2 receptor (4). ACE2-mediated viral entry is facilitated by serine proteases, most notably transmembrane protease serine 2 (TMPRSS2), which primes the SARS-CoV-2 spike glycoprotein (5). Initial infection of lung epithelia or alveoli allows SARS-CoV-2 to access the otherwise enclosed systemic circulation, subsequently pre-disposing multiple organs to potential infection. Multiple organs and tissues, such as the lungs, heart, kidneys, liver, and the vasculature, contain cells which co-express ACE2 and TMPRSS2, or other serine proteases (cathepsin B and cathepsin L1) (6–9).

Similar to other diseases caused by coronaviruses, the main transmission route of SARS-CoV-2 is *via* respiratory droplets and aerosolised particles (10) that are propelled into the air when a person speaks, coughs, shouts, sings, sneezes, or laughs. At the onset of the COVID-19 pandemic, the main symptoms were fever (98%), cough (76%), and myalgia or fatigue (44%) (11). Then, loss of sense of taste and smell, termed anosmia, became a symptom in March 2020 (12), with a large proportion of those reporting anosmia presenting with mild symptoms. Patients can then develop breathing difficulty within 1 week and the severely ill patients soon developed acute respiratory distress syndrome (ARDS), acute cardiac injury, secondary infections, or a combination, resulting in hospital admission and severe cases requiring mechanical ventilation in the ICU (11). Such patients typically exhibit an exaggerated immune response, or cytokine storm, that has become a hallmark of severe SARS-CoV-2 infection. Suppressing the pro-inflammatory nature of the disease is critical to improving patient morbidity and mortality rates and, therefore, developing and identifying viable therapeutic strategies is of urgent scientific importance. Transplantation of mesenchymal stem/stromal cells (MSCs) is one such potential therapy to combat COVID-19 induced inflammation and regeneration of damaged tissues.

The merits of MSCs are that they are multipotent stromal cells that can differentiate into a variety of cell types, including osteoblasts, chondrocytes, myocytes, and adipocytes that have their own characteristic structures and functions of specific tissues. They are typically found in the bone marrow, but have also been characterized in the adipose tissue, dental pulp, umbilical cord tissue, amniotic fluid, and heart (13). Mesenchymal stromal cells are easily accessible from various tissues, are free from ethical issues and have demonstrated no adverse outcomes in clinical trials. They have high proliferation rates, can be systemically administered, and possess key stem cell properties, such as multipotency (14, 15), in addition to being effective immunomodulators, collectively making MSCs a promising therapy in improving COVID-19 morbidity and mortality.

### Old Age, Being Male and CVD Co-morbidity—Significant Risk Factors for Mortality

Severity and high mortality from COVID-19 has been linked to old age, being male, cardiovascular disease (CVD), hypertension, and cardiometabolic disease including diabetes and obesity. A retrospective, multicentre cohort study by Zhou et al. (16) examined 191 patients, of whom 137 were discharged and 54 died in hospital. Of these patients, 91 (48%) had a comorbidity, with hypertension being the most common [58 (30%) patients], followed by diabetes [36 (19%) patients] and coronary heart disease [15 (8%) patients]. Multivariable regression analysis showed increasing odds of in-hospital death associated with older age [odds ratio (OR) 1.10, 95% CI 1.03–1.17, per year increase; *p* = 0.0043], higher Sequential Organ Failure Assessment (SOFA) score (5.65, 2.61–12.23; *p* < 0.0001), and D-dimer >1 μg/mL (18.42, 2.64–128.55; *p* = 0.0033) on admission. In univariable analysis, odds of in-hospital death was higher in patients with diabetes or coronary heart disease. Age, lymphopenia, leucocytosis, and elevated ALT, lactate dehydrogenase, high-sensitivity cardiac troponin I, creatine kinase, D-dimer, serum ferritin, IL-6, prothrombin time, creatinine, and procalcitonin were also associated with death (16).

In a retrospective case series involving 1,591 critically ill COVID-19 patients admitted from February 20 to March 18, 2020 in Lombardy, Italy, who required treatment in the ICU, the median (IQR) age was 63 (56–70) years and 1,304 (82%) were male. Of the 1,043 patients with available data, 709 (68%) had at least one comorbidity and 509 (49%) had hypertension. The second most common comorbidities were CVD [223 patients, 21% (95% CI, 19–24)] and hypercholesterolemia [188 patients, 18% (95% CI, 16–20%)]. ICU mortality was higher in those who were older (≥64 years). The prevalence of hypertension was higher among patients who died in the ICU (63%, 195 of 309 patients) compared with those discharged from the ICU (40%, 84 of 212 patients) [difference, 23% (95% CI, 15–32); *P* < 0.001] (17).

Emerging evidence strongly implicates COVID-19 as a vascular disease, with many COVID-19 positive patients purportedly developing cardiovascular complications, such as myocardial injury (18), cardiac arrhythmia (19) and thromboembolism (20, 21). Interestingly, cardiovascular complications have also been reported in patients with no underlying pathology, for instance with acute viral myocarditis (22, 23). Cardiovascular (CV) system involvement is associated with higher mortality rates and is largely indicated by elevated inflammatory biomarkers, including D-dimer, cardiac troponin (cTn), ferritin, and interleukin (IL)-6 (24). For further insight, readers are directed to our review on Vascular Manifestations of COVID-19 (25) in this series.

### Myocardial Damage: The Role of Cardiac Troponin and Other Relevant Markers

A number of studies show that a high proportion of COVID-19 patients exhibit elevated levels of cardiac damage biomarkers, such as cTn, with reports of up to 38% of patients testing positive for COVID-19 displaying high circulating levels of cTn (26). In comparison to COVID-19 patients with low cTn, those exhibiting high levels of cTn are hospitalized for longer requiring mechanical ventilation and admission to ICU, are at a significantly greater risk of developing ARDS and cardiac arrhythmias, and ultimately have a higher risk of mortality (27). In a study comparing clinical characteristics between survivors of COVID-19, and those who succumbed to the disease, researchers found that elevated levels of cTn were found in 77% of patients who subsequently died, compared to only 14% of patients who had survived (28). In addition, Guo et al. (29) showed that myocardial injury (elevated cTnT levels) was associated with worse outcome. Patients with underlying CVD are more likely to present with high cTn levels, with the poor prognosis for those with elevated levels further compounded if the patient had underlying CVD, compared to those without underlying CVD (69.4 vs. 37.5% mortality rate, respectively) (29). In the study by Zhou et al. (16) the highest OR for mortality in COVID-19 patients (*n* = 191) was for elevated cTn (>28 pg/mL, OR: 80.1) compared to other biomarkers, including circulating lymphocyte count (OR: 0.02) and D-dimer (OR: 20.04). It is also evident that throughout hospitalization, levels of cTn rise, and importantly, survivors showed no rise in this biomarker during the hospital stay, whereas patients with COVID-19 who died from complications, showed a steady upward rise in cTn until death (16). In another study, a significant predictor of mortality due to COVID-19 was the peak cTn during hospitalization, not the level measured upon admission (26), suggestive that risk stratification should include serial cTn measurements.

Besides cTn, other biomarkers, such as creatine kinase (CK), electrocardiographic (ECG) changes, and imaging might also reveal cardiac pathology in COVID-19 patients. Data acquired from multi-centers showed plasma lactate dehydrogenase and CK levels were correlated with COVID-19 severity and ICU admissions, reaching 26.1 and 70.5%, respectively (30). CK isoenzyme-MB (CK-MB), myohaemoglobin (MYO), and N-terminal pro-brain natriuretic peptide (NT-proBNP) are elevated above normal ranges in 3.7, 10.6, and 12.4% confirmed cases, respectively (31). When stratified by disease severity, patients with abnormal CK-MB, MYO, and NT-proBNP increased to 6.7, 26.7, and 33.3% respectively in the critical cases, underscoring underlying ischaemia and cardiac dysfunction. This is further supported by ECG findings characteristic of ischaemia, such as T-wave depression and inversion, ST depression, and presence of Q waves (18). In a case report, the presence of acute pulmonary embolism in COVID-19 was associated with right ventricular dilatation and dyskinesis on echocardiography, indicating that some patients develop ventricular hypertrophy (32).

### Immune Response to COVID-19: Healthy vs. Hyperactive

The immune response to COVID-19 can be split into a healthy antiviral immune response or a defective/overactive immune response. The latter has been linked to damage to the lungs and other organs, resulting in onset of severe illness. Initially, SARS-CoV-2 infection and destruction of lung cells switches on antiviral defenses triggering a local immune response. This includes recruitment of macrophages and monocytes to respond to the infection, interferons and release of cytokines and chemokines and primed adaptive T and B cell immune responses. In most cases, this process is capable of resolving the infection. However, in some cases, a dysfunctional immune response occurs, resulting in severe lung and multi-system damage, and possible failure (33).

In the healthy immune response, the innate antiviral defenses fight against the virus and virus-specific T cells can later eliminate the infected cells before the virus spreads. Neutralizing antibodies in these individuals can block viral infection, and phagocytic cells such as alveolar macrophages recognize neutralized viruses and apoptotic cells and clear them by phagocytosis. Altogether, these processes lead to clearance of the virus with minimal lung and multi-system damage, resulting in recovery (33).

In a defective immune response, there is a hyperactivation of the immune cells, with excessive infiltration of monocytes, macrophages and T cells, in the lungs. This causes overproduction of pro-inflammatory cytokines, the so-called “cytokine storm” or “cytokine release syndrome,” which eventually can lead to lung damage, pulmonary oedema and pneumonia. The resulting cytokine storm leads to widespread inflammation circulating to other organs, leading to multiple organ damage (33). Elucidating the mechanisms underlying the immune response to COVID-19 and the causes for the hyperactivation of the immune response are at the forefront of this exciting research area. Recently, Merad and Martin (34) reviewed how activated monocyte-derived macrophages leading to a dysregulated macrophage response contribute to the COVID-19 cytokine storm by releasing massive amounts of pro-inflammatory cytokines (34). Moreover, the biological and clinical consequences of the so-called cytokine storm are still largely unknown.

## Cytokine Storm in COVID-19

The term cytokine storm was first employed in describing the events modulating the onset of graft-vs.-host disease (35). Cytokine storms characterize a wide spectrum of infectious and non-infectious diseases. Since 2005, it was associated to the avian H5N1 influenza virus infection (36) and then infections with MERS and SARS, with an inflammatory milieu containing IL-1β, IL-6, and TNF-α being associated with worse disease outcomes (37). Now, severe COVID-19 disease caused by SARS-CoV-2 infection is also associated with a dysregulated and hyperactive systemic inflammatory response; a cytokine storm (38).

It was first reported that several pro-inflammatory cytokines and chemokines, including IL-2, IL-7, IL-10, CXCL10 (IP-10), CXCL8, CCL2 (MCP1), TNFα, and IFNγ were higher in the plasma of COVID-19 patients as compared to healthy controls. More importantly, among infected patients, IL-2, IL-7, IL-10, granulocyte colony- stimulating factor (G-CSF), macrophage inflammatory protein 1α (MIP1α), CXCL10, CCL2, and TNFα circulating concentrations (but not those of IFNγ) were found to be significantly higher in patients requiring admission to ICU and mechanical ventilation, compared to patients experiencing a less severe clinical course (11).

Chen et al. (39) characterized the immunological features of COVID-19 patients presenting with differing disease severity. Eleven patients with severe disease displayed significantly higher serum levels of IL-6, IL-10, and TNF-α and lower absolute numbers of T lymphocytes, CD4^+^T cells, and CD8^+^T cells as compared with 10 patients with moderate disease. Of note, severe cases were characterized by a lower expression of IFN-γ by CD4^+^T cells as compared with moderate cases (39). Likewise, analysis from Liu et al. (40) demonstrated significant decreases in the counts of T cells, especially CD8^+^ T cells, as well as increases in IL-6, IL-10, IL-2, and IFN-γ levels in the peripheral blood in the severe COVID-19 cases (*n* = 13) compared to those in the mild cases (*n* = 27), suggesting that disease severity is associated with significant lymphopenia and hyperinflammation.

Del Valle et al. (41) used a multiplex cytokine assay to measure serum IL-6, IL-8, TNF-α, and IL-1β in hospitalized COVID-19 patients (*n* = 1,484) upon admission to the Mount Sinai Health System in New York, USA. They showed that serum IL-6, IL-8, and TNFα levels at the time of hospitalization were strong and independent predictors of patient outcomes, with elevated inflammatory profile associated with reduced survival. Importantly, when adjusting for disease severity score, common laboratory inflammation markers, hypoxia and other vitals, demographics, and a range of comorbidities, IL-6 and TNF-α serum levels remained independent and significant predictors of disease severity and death (41).

In an elegant study, Lucas et al. (42) have identified that development of a maladaptive immune response profile was associated with severe COVID-19 outcome, and early immune signatures correlated with divergent disease trajectories. Through serially analyzing immune responses in peripheral blood in 113 COVID-19 patients with moderate (non-ICU) and severe (ICU) disease, they revealed an association between early, elevated cytokines and worse disease outcomes. Indeed, they observed a “core COVID-19 signature” shared by both moderate and severe groups of patients defined by the following inflammatory cytokines that positively correlated with each other; these included: IL-1α, IL-1β, IL-17A, IL-12 p70, and IFN-α. In severe patients, they observed an additional inflammatory cluster defined by: thyroid peroxidase (TPO), IL-33, IL-16, IL-21, IL-23, IFN-λ, eotaxin, and eotaxin 3. Interestingly, most of the cytokines linked to cytokine release syndrome, such as IL-1α, IL-1β, IL-6, IL-10, IL-18, and TNF-α, showed increased positive associations in severe patients. After day 10, in patients with moderate disease, these markers steadily declined. In contrast, severe patients maintained elevated levels of these core signature makers. Notably, additional correlations between cytokines emerged in patients with severe disease following day 10. Therefore, there were sharp differences in the expression of inflammatory markers along disease progression between patients who exhibit moderate vs. severe COVID-19 symptoms. Altogether, data showed a broad elevation of type-1, type-2, and type-3 signatures in severe cases of COVID-19, with distinct temporal dynamics and quantities between severe and moderate patients. Unsupervised clustering analysis of plasma and peripheral blood leukocyte data identified four immune signatures, representing (A) tissue repair growth factors, (B) type-2/3 cytokines, (C) mixed type-1/2/3 cytokines, and (D) chemokines involved in leukocyte trafficking that correlated with three distinct disease trajectories of patients. The immune profile of patients who recovered with moderate disease was enriched in tissue reparative growth factor signature (A), while the profile for those with worsened disease trajectory had elevated levels of all four signatures. Overall, results suggested that a multi-faceted inflammatory response is associated with late COVID-19 severity, which raises the possibility that early immunological interventions that target inflammatory markers predictive of worse disease outcome are preferred to blocking late-appearing cytokines.

Supporting the work of Lucas et al. (42) a recently published article has identified a core peripheral blood immune signature across 63 hospital-treated patients in London, UK with COVID-19. Specifically, among several changes in immune cells expressed at unusual levels in the blood of patients, the work identified a triad of IP-10 (CXCL10), IL-10, and IL-6 to correlate strongly with disease severity. Indeed, patients with COVID-19 who displayed measurably higher levels of IP-10 (CXCL10), IL-10, and IL-6 when first admitted to hospital went on to become more severely ill. The triad of cytokines was found to be a rigorous predictor of disease severity than commonly-used clinical indicators, including CRP, D-dimer, and ferritin (43).

As the COVID-19 cytokine storm is a multi-faceted inflammatory response, therapies that target this as a whole and those that enhance tissue repair (i.e., mesenchymal stem/stromal cells; MSCs) should be considered. Indeed, Lucas et al. (42) found IL-6 to be highly enriched in patients with severe disease. In fact, all ICU patients in their study, including the ones who succumbed to the disease, received Tocilizumab, an IL-6R blocking antibody. Positive outcomes have been reported with Tocilizumab treatment, including a reduction in an inflammatory-monocyte population associated with worse outcomes (44). However, as patients still succumbed to COVID-19, this highlights the need for combination therapy to block other cytokines highly represented in severe COVID-19 cases, including inflammasome-dependent cytokines and type-2 cytokines (42).

## The Effects of the COVID-19 Cytokine Storm

### On the Lungs Leading to Acute Respiratory Distress Syndrome (ARDS)

Acute respiratory distress syndrome (ARDS) is a form of hypoxaemic respiratory failure that is characterized by severe impairment of gas exchange and lung mechanics, with a high case fatality rate. Acute respiratory distress syndrome can come about through the severe widespread inflammatory injury present throughout the lungs, leading to a loss of vascular barrier integrity and likely promoting pulmonary oedema, thereby causing inflammation of endothelial cells (endothelialitis). Acute respiratory distress syndrome is a prominent feature in patients with severe COVID-19 infection (45, 46) and is the leading cause of mortality (47).

The precise pathophysiological mechanisms underlying ARDS in COVID-19 patients are not fully understood. However, alveolar macrophages are central to mediating the inflammation associated with ARDS (48), with the initial inflammatory stage involving alveolar macrophages interacting with lymphocytes (49) and epithelial cells (50), thereby augmenting the inflammatory response and accentuating tissue damage (51). Following initial stimulation, neutrophils and circulating macrophages are recruited to the lungs (activated by the pro-inflammatory cytokines), thereby triggering further inflammatory responses (52) equating to a positive feedback loop. These cells may disrupt the air–blood barrier by causing collateral tissue damage, particularly to airway epithelial cells and vascular endothelial cells, which express the ACE2 entry receptor for SARS-CoV-2; the damage of vascular endothelial cells may account for thrombotic microangiopathies (53). Furthermore, severe infection of the lung alveoli allows the SARS-CoV-2 virus and pro-inflammatory cytokine overload to enter the systemic circulation where it can infiltrate multiple organs, particularly since cells in many of them co-express ACE2 and TMPRSS2 (7, 8, 54).

In addition to the marked lung damage observed in COVID-19 infection, clinical cohort studies have revealed involvement of the kidneys (11, 16, 19, 30, 55, 56), liver (11, 30, 57, 58), gastrointestinal tract (11, 30, 59, 60), central nervous system (61, 62), and CV system (16, 18, 19, 63).

### Mitochondrial-Related Mechanisms

Mitochondria are essential for meeting the rise in energy demand required to fuel the immune system response and also for inducing immunomodulatory mechanisms, serving as a platform for host defense against RNA viruses such as SARS-CoV-2 (64, 65). The effects of SARS-CoV-2 infection upon mitochondrial respiratory capacity is a key consideration in the context of the host cytokine response. Mitochondrial respiratory capacity has been suggested to account for 10–30% of the variance in circulating leukocyte immune reaction across individuals, influencing the cytokine signature produced by leukocytes in response to lipopolysaccharide (LPS) administration (66). In particular, complex IV activity was positively correlated with LPS-stimulated IL-6 release (66). This is of particular interest in relation to SARS-CoV-2, whereby blood IL-6 has been identified as a predictor of patient fatality (47).

Aside from respiration, mitochondria are essential in host cell detection of RNA *via* pattern recognition receptors (PPRs), including cytosolic sensors retinoic acid-inducible gene 1 (RIG-1) and melanoma differentiation-associated protein 5 (MDA5) (67). These utilize the mitochondrial signaling protein MAVS (mitochondrial antiviral signaling protein), which recruits the E3 ligases TNF receptor associated factor 3 (TRAF3) and TRAF6, facilitating activation of interferon regulatory factors (IRFs) and NF-κB to induce antiviral genes. In this manner, MAVS activity coordinates the activation of a dominant antiviral mechanism, the type 1 interferon (IFN) pathway (64). SARS-CoV-2 open reading frame (Orf) 9b targets the translocase of outer mitochondrial membrane protein 70 (TOMM70), linking mitochondrial signaling to induction of the IFN pathway (68). The Orf9b of SARS-CoV-2 also localizes to the outer mitochondrial membrane, disrupting the MAVS signalosome (69) and impairing the host IFN response (69, 70). Other mitochondrial factors that may impact the IFN response include mitochondrial stress, whereby release of mtDNA into the cytosol is detected by the DNA sensor cGAS, which promotes STING-IRF3 signaling, potentiating IFN pathway signaling (71).

Inflammasomes, the multiprotein complexes providing a platform for the activation of pro-inflammatory caspase-1 culminating in cytokine release, are also mitochondrial-dependent. An example is NLRX1, a target of SARS-CoV-2 Orf9c (68). NLRX1 interacts with mitochondrial complex III, stimulating reactive oxygen species (ROS) production (72). ROS production from mitochondrial complexes I and III is known to mediate both innate and adaptive viral immune responses (73), impacting both MAVS and NF-κB signaling (72).

Pro-inflammatory cytokines are known to elicit metabolic alterations, with NF-κB and interleukin signaling impacting glucose control and glycolytic function. For instance, development of insulin resistance has been linked to IL-1 and IL-6 signaling in the context of type 2 diabetes mellitus (74). This is a key consideration in SARS-CoV-2, whereby poor blood glucose control has been associated with higher mortality in diabetic patients (75) and high glucose levels associated with viral replication in monocytes, with enhanced glycolytic capacity coinciding with raised IL-1β (76).

NF-κB mediated metabolic re-programming has been demonstrated in acute viral myocarditis (VM) (77, 78), a condition characterized by viral induced leukocyte infiltration and cardiac dysfunction. Case studies of acute VM have been reported in female COVID-19 patients (ages 21 and 43), resulting in substantial disruption to cardiac function in the absence of coronary artery disease (22, 23). Viral fulminant myocarditis, a syndrome on the clinical spectrum of acute myocarditis, has also been associated with death in SARS-CoV-2 patients suffering from cardiac injury (79).

In human and mouse models of VM, cardiac inflammation indicated through cytokine mediated NF-κB activation was linked to impaired expression of genes related to oxidative metabolism. This included downregulation of genes encoding mitochondrial regulatory proteins associated with biogenesis (PGC-1α, PGC1-1β, Tfam, and NRF-1) alongside regulators of β-oxidation (e.g., PPAR-α), tricarboxylic acid cycle and electron transport chain (ETC) function. This coincided with a fall in high energy phosphates and NAD levels and a shift toward anaerobic glycolysis, indicated through increased expression of glucose and lactate transporters and glycolytic enzymes (77). Together, this indicates that the inflammatory response associated with acute VM initiates reprogramming of cardiomyocyte energy metabolism away from oxidative metabolism and toward glycolysis. This culminated in an energy-starved status of the heart, the extent to which likely contributed to impaired cardiac function. NF-κB signaling has also been linked to impaired insulin signaling by stimulating phosphorylation of insulin receptor substrate-1, in turn inducing insulin resistance and cardiac dysfunction associated with VM (78). The metabolic implications of VM onset and resulting impairment of myocardial function are thus vital considerations in the pathophysiology of SARS-CoV-2 infection.

### On the Cardiovascular System

A number of case reports have demonstrated cardiac abnormalities in patients with COVID-19, including myocarditis, myo-pericarditis, electrocardiographic complications, cardiogenic shock, decompensated heart failure, and other histological/imaging complications, such as reduced left ventricular ejection fraction (LVEF) (80–85). Moreover, and as described previously, cross-sectional studies have consistently reported elevations in cardiac injury markers, such as cTn, NT-proBNP, and creatine kinase myocardial band (CK-MB) concentrations, with patients presenting with cardiac injury being at a higher risk of mortality, even after being adjusted for confounding variables such as age, pre-existing CVD, and ARDS (18). These data give strong evidence for cardiac complications associated with COVID-19, however, the mechanisms for these complications may not be solely the result of a direct viral infection of cardiac cells.

The CV system is also at high-risk as a result of indirect mechanisms, such as the cytokine storm. The cytokine storm is likely to induce cardiovascular damage through mechanisms related to endothelial dysfunction, atherosclerotic plaque instability/rupture, cardiomyocyte death, and myocarditis. The mechanisms of endothelial dysfunction within the COVID-19 population are not limited to elevations in pro-inflammatory cytokine concentrations and include direct viral infection of endothelial cells, angiotensin II (Ang II) hyperactivity, complement activation, and other elements of immune dysregulation, such as neutrophil extracellular trap (NET) formation. Indeed, evidence of SARS-CoV-2 viral structures have been observed in endothelial cells in various tissue beds (63), which may promote an imbalance between ACE2 and Ang II. Liu et al. (86) support this notion by demonstrating elevated plasma Ang II concentrations in patients with COVID-19. For a more in depth review of direct viral infection of endothelial cells, including Ang II hyperactivity, readers are directed to our recent review on the vascular manifestations of COVID-19 (25). Complement activation has been associated with microthrombosis in a small number of patients with COVID-19 (87) and NET formation has been correlated with COVID-19-associated ARDS (88). Both complement activation and NET formation are associated with pro-inflammatory responses. The complement system detects viral pathogens, thus contributing to the innate immune response to viral infections (89), whilst NETs have the ability to induce IL-1β secretion from macrophages and play a role in the development of atherosclerosis, causing endothelial damage and dysfunction (90, 91). Moreover, endothelial cells undergoing apoptosis have been shown to activate the complement system (92), which may further exacerbate cytokine secretion and promote microthrombosis. Therefore, it should be acknowledged that direct viral infection of endothelial cells, subsequent Ang II hyperactivity and the pro-inflammatory effects of complement activation and NET formation promote both direct and indirect perturbations to the cardiovascular system, whilst exacerbating the cytokine storm. Moving forward, the predominant focus of this section is to discuss the potential effects of the cytokine storm upon the cardiovascular system.

The cytokine storm is not only one of the predominant pathophysiological mechanisms of fulminant myocarditis (without evidence of viral infiltration) (93), which has been reported in patients with COVID-19, but inflammatory infiltration into endothelial cells has also been reported in histological studies (63, 94). Inflammatory infiltration into endothelial cells promotes endothelialitis, perturbing endothelial cell membrane function, loosening inter-endothelial junctions, and causing cell swelling (94, 95). Indeed, Varga et al. (63) showed endothelial cell death and dysfunction in patients infected with SARS-CoV-2, which facilitated the induction of endothelialitis in several organs, including cardiac tissue, as a direct consequence of viral involvement and of the host inflammatory response.

The presence of endothelialitis demonstrates the activation of endothelial cells, promoting the expression of cell-surface adhesion molecules and thus the binding of inflammatory cells to the endothelium (96, 97). These pathophysiological consequences promote vascular hyperpermeability. Disruption of inter-endothelial junctions cause endothelial cells to be “pulled apart,” thus resulting in inter-endothelial gaps (95, 98), denoting cytoskeletal alterations to the endothelium. Moreover, this cytokine storm-induced endothelial dysfunction pre-disposes the CV system to a pro-coagulant state, promoting thromboembolic events, which has been linked to higher disease severity, and higher instances of mortality (99). Interestingly thrombin exposure, coupled with an elevation in the influx of Ca^2+^ promotes elevations in endothelial cell permeability which can be induced by an increase in TNF-α expression (100, 101).

Elevations in cytosolic Ca^2+^ influx into endothelial cells is a pivotal step in the disruption to inter-endothelial junctions and thus the progression to increased vascular permeability (101, 102). A determinant of this increased Ca^2+^ influx is the upregulation of transient receptor potential channels, which is induced *via* TNF-α (100), causing a destabilization of microtubules (103). Evidence supports the notion of a cytokine-induced hyperpermeability response of the vasculature, with Tinsley et al. (104) demonstrating the role of cytokine (TNF-α, IL-1β, and IL-6) induced-vascular hyperpermeability through a protein kinase C (PKC) and myosin light chain kinase (MLCK) dependent mechanism in cultured rat heart microvascular endothelial cells. Moreover, the authors replicated these findings *in vivo* using a coronary ischemia/reperfusion (I/R) rodent model of heart failure, demonstrating TNF-α increases endothelial permeability in a PKC and MLCK dependent manner (104). Therefore, translating this to COVID-19 pathophysiology, cytokine storm induced Ca^2+^ influx into endothelial cells may be a contributing mechanism underpinning the disruption to inter-endothelial junctions and the promotion of vascular permeability. Furthermore, the cytokine-induced stimulation of PKC and MLCK may promote direct damage to cardiac tissue, which may pose significant deleterious effects upon patients with pre-existing CVD, a common comorbidity in the more severe COVID-19 population (105).

Histological studies in pulmonary vasculature have indicated endothelialitis, with unexpected observations of intussuseptive angiogenesis. In this study (94), the degree of intussuseptive angiogenesis was associated with the duration of hospitalization. Whilst hypoxia may be a contributing mechanism, the authors concluded the predominant mechanism was likely the presence of endothelialitis and thrombosis (94). Intussuseptive angiogenesis is the formation of intravascular vessel formation, through non-sprouting mechanisms, commonly observed as “pillar” formation within the vasculature (106), which can significantly alter the microcirculation, and can be triggered by extraluminal processes, including inflammation (107). Inflammatory-mediated intussuseptive angiogenesis has been demonstrated previously in murine models of colitis, suggesting this is an adaptive response to prolonged inflammation (108). This provides further evidence of the perturbations to the vasculature caused by the cytokine storm in COVID-19. The promotion of intussuseptive angiogenesis as an adaptive response to vascular damage, has also been shown to accelerate fibrotic neovascularisation (109).

Inflammatory environments also promote the generation of ROS which can result in damage and dysfunction of the vasculature. ROS act as signaling molecules to defend against oxidative stress by promoting the upregulation of antioxidant mechanisms, however, high concentrations of ROS can activate endothelial cells and inhibit normal endothelial functioning. Cytokines, such as TNF-α, have been shown to interact with the ETC and stimulate the release of mitochondrial-derived ROS, such as hydrogen peroxide (110) and superoxide (111). Moreover, in response to infections, inflammatory cytokines, such as TNF-α and IL-1β, coming into contact with endothelial cells induce NAD(P)H oxidase-derived ROS (112, 113). The generation of excessive ROS elevates superoxide anion production, which can degrade nitric oxide (NO), lead to the formation of other free radicals, such as peroxynitrite, and thus result in endothelial cell dysfunction and apoptosis (96, 114, 115). Therefore, it is likely that the cytokine storm experienced in patients with COVID-19 will promote the elevation in ROS and result in oxidative stress, which is a key mechanism of endothelial dysfunction in hypertension (116) and CVD (117). Elevations in ROS also act as secondary inflammatory signals, which has been shown to induce the secretion of pro-inflammatory cytokines, such as IL-1β, TNF-α, and IL-6 (118). Therefore, this creates a vicious cycle of cytokine-induced oxidative stress and ROS-induced pro-inflammatory cytokine signaling, secondary to the COVID-19 hyper-activation of the immune response.

Inflammatory cytokines do not just alter endothelial structure and function. Cytokines such as TNF-α, IL-1β, and IL-6 promote vascular smooth muscle cell (VSMC) proliferation from the media to the intima of the vasculature, which results in the secretion of extracellular matrix proteins within, and thus expanding the intima in pathological conditions, such as atherosclerosis (119). Moreover, in human coronary VSMCs, IL-1β has been shown to stimulate an upregulation in Rho-kinase, *via* a PKC-dependent mechanism, which may contribute to medial thickening and the atherogenic environment (120). Interestingly, this can also be stimulated by an upregulation in angiotensin II, which has been noted within the COVID-19 literature if infected cells experience a downregulation of ACE2 expression (121), which will also contribute to the pro-inflammatory environment experienced in patients with COVID-19. Activation of RhoA can also be stimulated by TNF-α which has been shown to promote endothelial cell permeability in cultured human umbilical vein endothelial cells (HUVECs) (122). These pathophysiological processes are shared with thrombosis, which is a common manifestation in patients with severe COVID-19 (99). Combined with damage to endothelial cells contributing to the apparent “COVID-19 coagulopathy” (123), VSMC proliferation, stimulated by various cytokines, may contribute to the high instance of coagulation derangements and thromboembolic events observed in patients with severe COVID-19.

Whilst the COVID-19 induced cytokine storm can pre-dispose the CV system to damage and progression of pre-existing cardiovascular comorbidities, perturbations to vascular cells may also contribute to the overexpression of pro-inflammatory cytokines. Both endothelial cells and VSMCs secrete pro-inflammatory cytokines when either damaged or undergoing apoptosis. Expression of cell-surface adhesion molecules and certain cytokines, such as IL-8, on the surface of endothelial cells induce a pro-inflammatory phenotype and the recruitment of blood monocytes which induce the secretion of pro-inflammatory cytokines, such as TNF-α and IL-1β (124). Moreover, under atherogenic conditions, VSMCs have been shown to also adopt a pro-inflammatory phenotype, promoting the secretion of IL-6 and IL-8, along with cell-surface adhesion molecules, such as vascular cell adhesion molecule 1 (124, 125). Therefore, both endothelial cells and VSMCs, once damaged, may switch to a pro-inflammatory phenotype and thus propagate the expression of pro-inflammatory cytokines.

Whilst there is a plethora of evidence which suggests that the cytokine storm experienced in COVID-19 patients may promote damage to the vasculature, sustained inflammation directly contributes to progressive cardiomyocyte apoptosis. Elevated TNF-α levels seen in a variety of clinical conditions including COVID-19, drives cardiomyocytes to apoptosis (126, 127). TNF-α can induce cardiomyocyte apoptosis directly, *via* the TNF receptor, or indirectly, through stimulation of NO production or ROS, which in turn is induced by pro-inflammatory cytokines such as IL-1, IL-6, TNF-α, and IFN-7 (128). High levels of cTn are reflective of cardiomyocyte death and injury, and as stated earlier, are associated with COVID-19 disease severity and mortality (16).

In the heart, the acute inflammatory response can expand tissue damage and prolonged inflammation leads to accentuated adverse remodeling. Indeed, pro-inflammatory cytokines and upregulated monocytes/macrophages can inhibit cardiac repair, which is dependent on timely suppression and resolution of pro-inflammatory signaling. Activation of IL-1 signaling induces cytokine expression, promotes matrix-degrading properties, suppresses fibroblast proliferation and inhibits transdifferentiation of fibroblasts into myofibroblasts, altogether delaying activation of a reparative response (129). Moreover, a severe or prolonged reparative response is associated with pathological scarring and fibrosis (130).

The full extent of cardiovascular cell dysfunction and death, induced by the cytokine storm in COVID-19, is yet to be fully elucidated. This section provides evidence of the potential effects and mechanisms of the COVID-19 cytokine storm on the cardiovascular system. It is likely that cardiomyocyte and vascular cell damage and dysfunction, as well as mitochondrial-related mechanisms play a role in the progression of COVID-19 and in the pathogenesis of cardiovascular injury in COVID-19. The induction of ROS generation and the ensuing oxidative stress, coupled with vascular cell secretion of pro-inflammatory cytokines further propagates the inflammatory environment and exaggerated immune response in patients with COVID-19, promoting disease progression and multi-organ dysfunction. Moreover, cardiac and vascular cell dysfunction pre-disposes the CV system to a pro-inflammatory and pro-atherogenic state and thus increases the risk of serious cardiac events. Therefore, suppression of the cytokine storm, is key for improving patient outcomes with COVID-19, whilst also protecting the CV system. One such therapy is transplantation of mesenchymal stem/stromal cells (MSCs).

## MSCs as a Therapy for Severe COVID-19 Patients

### Immunomodulatory Role of MSCs

An important function of MSCs is that they have powerful immunomodulatory properties, possessing natural abilities to detect changes in their environment such as inflammation. Mesenchymal stromal cells can both directly and indirectly stimulate immunomodulation by interacting with immune cells and releasing various anti-inflammatory cytokines *via* paracrine effects, respectively (131). Functional alterations to dendritic cells, monocytes, macrophages, regulatory T-cells (Tregs), and B-cells underpin MSCs' immunomodulatory capacity, whilst also through cell-to-cell interaction mechanisms (13). Once systemically administered, a significant portion of MSCs accumulate within the lungs, which can promote anti-inflammatory effects, thus improving the lung microenvironment and potentially restoring vascular barrier integrity and reducing oedema; whilst also promoting endogenous repair and regeneration mechanisms to reduce (or prevent further) fibrosis of the lung (132, 133).

Animal models of ARDS lung injury due to influenza virus have shown that infection by this and related viruses causes ion channel transporter abnormalities which causes fluid secretion, a major cause of the pulmonary oedema in the lungs of infected individuals. In such animal models, MSCs prevent or reduce the secretory effect of influenza virus on lung alveolar cell ion channels, and when administered intravenously in aged animals have resulted in increased oxygenation, improved respiration, reduction in pro-inflammatory cytokines, and an increase in survival (134).

Mesenchymal stromal cells are well-known to respond to the inflammatory environment with multimodal activity resulting in sustained anti-inflammatory effects; conversion of Th17 cells to anti-inflammatory FOXP3 Treg cells by MSC-secreted transforming growth factor (TGF) β1 and the essential presence of CCL18 producing type-2 anti-inflammatory macrophages from differentiated pro-inflammatory monocytes (135). They are known to dampen the innate immune response to insult (such as acute lung injury, burn injuries) or infection *via* preventing neutrophil infiltration into injured/infected sites (136–139) or *via* shifting the phenotype of macrophages from an M1 to M2 anti-inflammatory phenotype (140). Specifically the MSCs appear to reduce inflammation *via* reducing macrophage secretion of neutrophil chemoattractant proteins CXCL1, CXCL2 (137, 141) as a result of activation of phosphorylation of p38 MAPK (141) and greater IL-10 release (137), dampened production of IL-6 and TNF-α (137, 138), and suppression of reactive oxygen species production by neutrophils (142, 143). Together this contributes toward a shift from a pro- to an anti-inflammatory environment and is an essential part of the immunomodulatory function of MSCs as this helps prevent against autoimmunity (13), as demonstrated in MSC-treated graft vs. host disease (144).

Mesenchymal stromal cells can also induce local and systemic immunomodulatory responses independently of the cytokine storm. For instance, MSCs can prevent the infiltration of cells of the innate immune system, thereby indirectly reducing the secretion of inflammatory cytokines. In a murine model, BM-MSCs reduced CD45^+^ cells and neutrophil populations in the mucosa *via* release of tumor necrosis factor-induced protein 6 (TSG-6) (145). Both MSCs and TSG-6 induced the expansion of regulatory macrophages, expressing IL-10 and inducible nitric oxide synthase (NOS), and increased the population of FOXP3CD45^+^ cells. Interestingly, TSG-6 was associated with MSC-mediated depletion of corneal, splenic, and peripheral blood CD11b^+^ monocytes/macrophages in a model of inflammatory corneal neovascularization (146). In addition to TSG-6, MSCs can also release other bioactive molecules that promote protective responses in innate immune cells, including kynurenic acid (147), spermine (148, 149), and lactate (150). Adaptive immune cells, such as T and B cells, are also direct targets of MSCs. Following transplantation, MSCs form aggregates with B and T cells, stimulating the production of FOXP3 and IL-10 (145). Mesenchymal stromal cells directly inhibit the activation of cytotoxic CD8^+^ T-cells *via* downregulation of CD25, CD38, and CD69 (151). In B cells, MSCs downregulate chemotactic properties, with no effect on costimulatory molecules or cytokine production (152). Mesenchymal stromal cell-mediated indoleamine 2,3-dioxygenase signaling promotes the survival and proliferation of CD5^+^ Bregs (153). There are also data to suggest that MSCs could act *via* extracellular vesicles and exosomes to modulate innate and adaptive immunity (154, 155). The immunoregulatory mechanisms of mesenchymal stem and stromal cells in inflammatory disease are reviewed in (156).

Consequently, on the basis of these and other studies with MSCs in animal models, clinical investigators have postulated that human MSCs should be effective in the pathology of human ARDS (157). Indeed in a report of allogeneic MSCs in ARDS patients, a single low dose of cells (2 million cells/kg/BW) achieved rapid reduction in inflammatory cytokines and efficacy in influenza-related ARDS which was otherwise refractory to conventional supportive therapy (158). For further insight on the therapeutic potential of cell therapy to treat ARDS readers are directed too (159, 160).

The systemic redistribution of MSCs have the ability to target other organs that are damaged. As multi-organ damage is a common manifestation in patients with severe COVID-19, this makes MSCs an attractive therapy to combat not only lung damage, but also damage observed in other organs, such as the heart. Therefore, the use of MSCs to modulate the immune response, avoiding, preventing or attenuating the cytokine storm leading to multi-organ failure may be the key for the treatment of COVID-19 infected patients.

### Use of MSCs to Treat COVID-19

Table 1 summarizes the published clinical studies thus far using MSCs as a therapy to treat COVID-19. Table 2 summarizes the ongoing, registered clinical trials using MSCs as a therapy to treat COVID-19. For review articles on the rationale and treatment of COVID-19-related ARDS using MSCs, readers are directed to Moll et al. (165) and Can and Coskun (166).

**Table 1:** Summarisation of clinical studies and ongoing clinical trials assessing the therapeutic benefit of MSC transplantation in patients with COVID-19, including studies assessing the therapeutic potential of MSCs in patients with acute respiratory distress syndrome (ARDS), without COVID-19.

**Citation**	***N***	**Subjects**	**MSC source and dose**	**MSC timing**	**Recipient site**	**Results**
Leng et al. (133)	MSC transplant: *n* = 7; CON: *n* = 3	COVID-19 pneumonia	Clinical grade ACE2^−^ MSCs at 1 × 10^6^ cells/kg	The time when symptoms and/or signs were still getting worse, even as the expectant treatments were being conducted	Systemic	- ↑ IL-10 vs. CON - ↓ TNF-α vs. CON - ↔ IP-10 - Trend for ↑ VEGF vs. CON - Inflammation, AAT, MYO and CK reduced in critically severe patient with a reduction in ground-glass opacity and pneumonia infiltration
Liang et al. (161)	Case study	Critical COVID-19	Allogenic hUCMSCs at 5 × 10^7^ cells 3 times	Admitted 2 days after symptoms onset and MSCs were transplanted on the 9, 12, and 15th days after admission. In combination with antibiotics and thymosi*n* α1	Systemic	No side effects were observed. After 2nd administration: - ↓ Bilirubin, WBC and neutrophil count, CRP and ALT/AST - ↑ lymphocyte count - ↑ CD3^+^, CD4^+^, and CD8^+^ T cells - Trachea cannula removed After 3rd administration: - Pneumonia relieved - Removed from ICU 2 days following - Negative throat swab
Zhang et al. (162)	Case study	COVID-19 pneumonia - History of diabetes	Wharton's jelly-derived hUCMSCs at 1 x 10^6^ cells/kg	Admitted 5 days after symptoms onset and MSCs were transplanted on the 17^th^ day of admission	Systemic	Post-transplant: - COVID-19 symptoms disappeared 2 to 7 days - ↓ Ground glass opacity and pneumonia infiltration day 6 - ↑ CD3^+^, CD4^+^ & CD8^+^ T cells - ↓ CRP, IL-6 & TNF-α
Chen et al. (163)	MSC transplant: *n* = 17; CON: *n* = 44	H7N9-induced ARDS	Allogenic menstrual-blood-derived MSCs at 1 × 10^6^ cells/kg	3 patients treated with 3 infusion at the early stage of infection; 6 patients were treated with 3 infusions at the late stage of infection; 8 patients accepted 4 infusions of at late stage of infection	Systemic	At admission: - No differences, except ↓ PCT vs. CON At discharge: - ↑ mortality rate of CON - ↓ PCT, ALT, sCr, CK, PT, and D-dimer vs. CON At follow-up (5 year; *n* = 4): - ↑ Hb - ↓ PT
Sengupta et al. (164)	*N* = 23	COVID-19: cohort a (mild COVID-19): *n* = 1; cohort b (hypoxaemia and COVID-19): *n* = 20; cohort c (intubated COVID-19): *n* = 3	Bone-marrow derived MSCs exosome agent—ExoFlow-−15 mL	Not specified	Systemic	−71% patients recovered and/or were discharged after 5.6 days post-infusion - 13% remained critically ill - 16% died - 80% improved PaO_2_/FiO_2_ ratio within 3 days - ↓ CRP, ferritin and D-dimer on day 5 -↑ CD3^+^, CD4^+^, and CD8^+^ T cells on day 5

*CON, control; ACE2, Angiotensin converting enzyme 2; IL-10, Interleukin-10; TNF-α, Tumor necrosis factor α; IP-10, Interferon gamma-induced protein 10; VEGF, Vascular endothelial growth factor; AST, Aspartate amino transferase; MYO, Myoglobin; CK, Creatine kinase; hUCMSC, human umbilical cord mesenchymal stem cells; WBC, white blood cell; CRP, C-reactive protein; ALT, Alanine aminotransferase; ICU, intensive care unit; ARDS, Acute respiratory distress syndrome; PCT, Procalcitonin; sCr, serum creatinine; PT, Prothrombin time*.

**Table 2:** List of registered, ongoing, clinical trials using mesenchymal stem/stromal cells (MSCs) as a therapy to treat COVID-19.

**Clinical trials number**	**Participants**	**MSC source**	**Outcomes**
NCT04371393 (USA)	Target: *N* = 300	MSCs (Remestemcel-L) at 2 × 10^6^ cells/kg administered twice during first week (second infusion 4 days following first) plus standard care vs. placebo (Plasma-Lyte) (second infusion 4 days following first) plus standard care	- All-cause mortality - SAEs - No. of days off mechanical ventilation - Resolution/improvement of ARDS - Length of stay - Clinical improvement scale - Hs-CRP, IL-6, IL-8, TNF-α
NCT03042143 (Northern Ireland)—REALIST trial	Target: *N* = 75	Single infusion of human umbilical cord derived CD362 enriched MSCs at maximum tolerable dose from phase I (dose escalation pilot study) plus standard care vs. placebo (Plasma-Lyte) plus standard care	- Oxygenation index - SAEs - SOFA - Respiratory compliance - P/F ratio - Driving pressure - Extubation and reintubation - Ventilation free days - Length of ICU/hospital stay - Mortality
NCT04444271 (Pakistan)	Target: *N* = 20	Bone marrow derived MSCs at 2 × 10^6^ cells/kg on day 1 and 7 plus standard care vs. saline injection plus standard care	- Survival - No. oxygen support days - Time to negative nCoV test - CT scan - No. days to discharge
NCT04416139 (Mexico)	Target: *N* = 10	Umbilical cord derived MSCs from De bank Laboratory at 1 × 10^6^ cells/kg (no control group—data compared to controls treated in a previous trial)	- PaO_2_/FiO_2_ ratio - HR and RR - Body temperature - Leukocyte, lymphocyte, and platelet counts - PCT, fibrinogen, D-dimer, ferritin - CRP, TNF-α, IL-1, IL-10, IL-6, IL-17 - VEGF - T-cell analysis (CD4^+^ and CD8^+^) - NK and dendritic cells - SAEs - CT scan - nCoV-test
NCT04429763 (Colombia)—CELMA	Target: *N* = 30	Umbilical cord derived MSCs at 1 × 10^6^ cells/kg plus standard care vs. placebo (not stated) plus standard care control	- NEWS scale - Time to hospital discharge - Respiratory function - Inflammatory markers - Hematological and renal assessments
NCT04315987 (Brazil)	Target: *N* = 90	NestaCell MSCs at 2 × 10^7^ cells/kg on days 1, 3, 5, and 7 plus standard care vs. placebo (not stated) on days 1, 3, 5, and 7 plus standard care	- Change in clinical condition - Mortality - SpO_2_ - PaO_2_/FiO_2_ ratio - T-cell analysis (CD4^+^ and CD8^+^) - SAEs - Blood count and cardiac, hepatic, and renal profiles
NCT04366323 (Spain)	Target: *N* = 26	Allogenic and expanded adipose tissue derived MSCs at 8 × 10^6^ cells × 2 (no control group)	- Safety of administration (SAEs) - Efficacy of administration
NCT04456361 (Mexico)	Target: *N* = 9	Wharton's jelly derived MSCs at 1 × 10^8^ cells/kg (no control group)	- SpO_2_ - PaO_2_/FiO_2_ ratio - Ground glass opacity and pneumonia infiltration - LDH, CRP, D-dimer, and Ferritin
NCT04366271 (Spain)	Target: *N* = 106	Undifferentiated allogenic umbilical cord MSCs (dose not stated) vs. standard care	- Mortality due to lung involvement - All-cause mortality - Days without mechanical ventilation - Days without vasopressors - Negative nCoV-test - SAEs
NCT04252118 (China)	Target: *N* = 20	MSCs (source not stated) at 3 × 10^7^ cells at day 0, 3, and 6 vs. standard care	- CT scan - SAEs - Pneumonia evaluation - Mortality - T-cell analysis (CD4^+^ and CD8^+^) - AAT, CRP, and CK
NCT04313322 (Jordan)	Target: *N* = 5	Wharton's jelly derived MSCs at 1 × 10^6^ cells/kg for 3 doses, spaced 3 days apart (No control group)	- Alleviations of symptoms - CT scan - Negative nCoV-test
NCT04336254 (China)	Target: *N* = 20	Allogenic human dental pulp MSCs at 3 × 10^7^ cells at day 1, 4, and 7 vs. saline control at day 1, 4, and 7	- TTCI - CT scan - Immune function markers - Time for negative nCoV-test - Blood count and classification - SpO_2_ - RR - Body temperature - SAEs - CRP
NCT04346368 (China)	Target: *N* = 20	Bone marrow derived MSCs at 1 × 10^6^ cells/kg at day 1 vs. standard care	- PaO_2_/FiO_2_ ratio - SAEs - Clinical outcome - No. days in hospital - CT scan - Changes in viral load - T-cell analysis (CD4^+^ and CD8^+^) - Mortality - CRP
NCT04288102 (China)	Target: *N* = 100	Umbilical cord derived MSCs at 4 × 10^7^ at day 0, 3, and 6 vs. saline control at day 0, 3, and 6	- Pneumonia evaluation - Time to clinical improvement - PaO_2_/FiO_2_ ratio - Days on oxygen therapy - SpO_2_ - 6-min walk test - Lymphocyte counts - Cytokine/chemokine assessment - SAEs - All-course mortality
NCT04273646 (China)	Target: *N* = 48	Umbilical cord derived MSCs at 0.5 × 10^6^ cells/kg at day 1, 3, 5, and 7 plus standard care vs. saline control at day 1, 3, 5, and 7 plus standard care	- Pneumonia evaluation - SAEs - Survival - Organ failure assessment - CRP and Procalcitonin - Lymphocyte count - T-cell analysis (CD3^+^, CD4^+^, and CD8^+^) - CD4^+^/CD8^+^ ratio
NCT04339660 (China)	Target: *N* = 30	Umbilical cord derived MSCs at 1 × 10^6^ cells/kg vs. saline control	- TNF-α, IL-1β, IL-6, TGF-β, IL-8, PCT, CRP - SpO_2_ - Mortality - CT scan - Blood count recovery time - Duration of respiratory symptoms - Negative nCoV-test
NCT04382547 (Belarus)	Target: *N* = 40	Allogenic pooled olfactory mucosa derived MSCs (dose not stated) vs. standard care control	- nCoV-test - SAEs
NCT04457609 (Indonesia)	Target: *N* = 40	Umbilical cord derived MSCs at 1 × 10^6^ cells/kg with Oseltamivir and Azithromycin vs. standard care with Oseltamivir and Azithromycin	- Clinical improvement markers - General laboratory outcomes - PCT, bilirubin, D-dimer, and fibrinogen - Troponin and NT-proBNP - LIF, IL-6, IL-10, ferritin, CXCR3 - T-cell analysis (CD4^+^, CD8^+^, and CD56^+^) - VEGF - CT scan
NCT04352803 (USA)	Target: *N* = 20	Autologous adipose derived MSCs at 0.5 × 10^6^ cells/kg vs. standard care control	- SAEs - Progression and time to/on mechanical ventilation - Length of hospital stay - All-cause mortality
NCT04490486 (USA)	Target: *N* = 21	Umbilical cord derived MSCs at 1 × 10^8^ cells on day 0 and 3 vs. 1% human serum albumin in Plasmalyte A on day 0 and 3	- SAEs - Inflammatory markers - COVID-19 viral load - SOFA score - Electrolyte levels - LDH - No. ICU discharges - Vasoactive agent use - Mortality - Immune markers - CT scan
NCT04522986 (Japan)	Target: *N* = 6	Adipose derived MSCs at 1 × 10^8^ cells once a week for 4 weeks (no control group)	- SAEs
NCT04461925 (Ukraine)	Target: *N* = 30	Placenta derived MSCs at 1 × 10^6^ cells/kg once every 3 days for 3 infusions vs. standard care control	- PaO_2_/FiO_2_ ratio - Length of hospital stay - Mortality - CRP - CT scan - Duration of respiratory symptoms - Blood count recovery time
NCT04362189 (USA)	Target: *N* = 100	Allogenic adipose tissue derived MSCs (Hope Biosciences) at 1 × 10^6^ cells/dose at day 0, 3, 7, and 10 vs. saline control at day 0, 3, 7, and 10	- IL-6, CRP, TNF-α, and IL-10 - Oxygenation - RTRA - ECG assessment - Routine blood assessments - Cardiac, hepatic, and renal assessment - Blood count - Platelets, Prothrombin time, D-dimer, and INR - Immune markers - SAEs - Chest X-ray - CT scan - Negative nCoV-test
NCT04371601 (China)	Target: *N* = 60	Umbilical cord derived MSCs at 1 × 10^6^ cells/kg once every 4 days for 4 infusions vs. standard care control	- PaO_2_/FiO_2_ ratio - TNF-α and IL-6 - Immune markers - CRP and calcitonin
NCT04348461 (Spain)	Target: *N* = 100	Allogenic expanded adipose tissue derived MSCs at 1.5 × 10^6^ cells/kg vs. standard care control	- Efficacy of administration of MSCs - SAEs
NCT04452097 (USA)	Target: *N* = 9	Umbilical cord derived MSCs (3 groups): - Low dose: 0.5 × 10^6^ cells/kg - Middle dose: 1 × 10^6^ cells/kg - High dose: 1.5 × 10^6^ cells/kg	- SAEs - TEAEs - Selection of appropriate dose for Phase II trial
NCT04494386 (USA)	Target: *N* = 60	Umbilical cord lining derived MSCs at 1 × 10^6^ cells/dose vs. saline control—either a single dose or 2 doses separated by 48 h	- DLT - SAEs - Berlin definition of ARDS - SpO_2_ and PaO_2_/FiO_2_ ratio - No. of VFDs - Blood count - Routine blood assessments - BUN and urinalysis - AAT
NCT04345601 (USA)	Target: *N* = 30	MSCs (source not specified) at 1 × 10^8^ cells vs. standard care control	- SAEs - Change to clinical status
NCT04377334 (Germany)	Target: *N* = 40	Allogenic bone marrow derived MSCs (dose not stated) vs. standard care control	- Lung injury score - D-dimer - Pro-resolving lipid mediators - Phenotype of immune cells - Cytokine and chemokine analysis - Survival - Extubation - Lymphocyte subpopulation - Complement molecules - SARS-CoV-2 specific antibody
NCT04390139 (Spain)	Target: *N* = 30	Wharton's jelly derived MSCs at 1 × 10^6^ cells/kg on day 1 and 3 vs. placebo (not stated) on day 1 and 3	- All-cause mortality - SAEs - Need for mechanical ventilation - No. of VFDs - PaO_2_/FiO_2_ ratio - SOFA index - APACHE II score - Duration of hospitalization - Immune response - Feasibility of MSCs - nCoV-test - LDH, D-dimer, and ferritin - Subpopulations of lymphocytes and immunoglobins - *In vitro* response of receptor lymphocytes
NCT04392778 (Turkey)	Target: *N* = 30	MSCs (source not stated) at 3 × 10^6^ cells/kg on day 0, 3, and 6 to COVID-19 patients with a ventilator vs. saline control on day 0, 3, and 6 to COVID-19 patients with a ventilator vs. standard care control to COVID-19 patients without a ventilator	- Clinical improvement - CT scan - Negative nCoV-test - Blood tests
NCT04467047 (Brazil)	Target: *N* = 10	MSCs (source not stated) at 1 × 10^6^ cells/kg (safety and feasibility study)	- Survival - CRP - Length of hospital stay - PaO_2_/FiO_2_ ratio - Liao's score (2020) - CT scan - Negative nCoV-test
NCT04398303 (USA)	Target: *N* = 70	Allogenic umbilical cord derived MSCs at 1 × 10^6^ cells/kg vs. MSC conditioned media at 100 ml vs. placebo (MEM-α) at 100 ml	- Mortality - No. of VFDs - No. of days on O_2_ therapy - No. of ICU-free days - Pulmonary function - Berlin criteria score
NCT04437823 (USA)	Target: *N* = 20	Umbilical cord derived MSCs at 0.5 × 10^6^ cells/kg on day 1, 3, and 5 vs. standard care control	- SAEs - CT scan - Negative nCoV-test - SOFA score - Mortality - Clinical respiratory changes
NCT04269525 (China)	Target: *N* = 16	Umbilical cord derived MSCs at 3.3 × 10^7^ cells on day 1, 3, 5, and 7	- PaO_2_/FiO_2_ ratio - Mortality - Length of hospital stay - nCoV PCR and antibody-test - Lung imaging - WBC and lymphocyte count - PCT - IL-2, IL-4, IL-4, IL-6, IL-10, TNF-α, γ-IFN, and CRP - NK cells - T-cell analysis (CD4^+^, CD8^+^)
NCT04447833 (Sweden)	Target: *N* = 9	Allogenic bone marrow derived MSCs at 1 × 10^6^ cells/kg (*n* = 3) and 2 × 10^6^ cells/kg (*n* = 6)	- SAEs - All-cause mortality - Leucocytes and thrombocytes - CRP - Prothrombin - Creatinine - AST and AAT - NT-proBNP - Blood pressure - Body temperature - Efficacy for MSC use - Lung function - 6-min walk test - Quality of life assessment - Blood biomarkers - Sensitisation test
NCT04491240 (Russia)	Target: *N* = 90	Inhalation of MSC exosomes at 0.5–2 × 10^10^ nanoparticles for COVID-19 patients (*n* = 30) and SARS-CoV-2 pneumonia patients (*n* = 30) vs. inhalation of solution free placebo (*n* = 30)—inhalation twice a day for 10 days	- SAEs - TTCI - Blood gases - SpO_2_ - Chest imaging
NCT04333368 (France)	Target: *N* = 40	Umbilical cord Wharton's jelly derived MSCs at 1 × 10^6^ cells/kg at day 1, 3, and 5 vs. placebo (NaCl) control at day 1, 3, and 5	- PaO_2_/FiO_2_ ratio - Lung injury score - Mortality - No. of VFDs - Use of sedatives - Use of neuromuscular blocking agent - ICU-acquired weakness - SAEs - Quality of life at 1 year - Cytokine analysis - Anti-HLA antibodies
NCT04466098 (USA)	Target: *N* = 30	Thawed product containing MSCs (source not stated) at 300 × 10^6^ cells 3 times separated by 48 h vs. placebo (dextran and human serum albumin) control 3 times separated by 48 h	- SAEs - Inflammatory markers - PaO_2_/FiO_2_ ratio - Mean airway, peak and plateau pressure - PEEP - Mortality - No. of ICU free days - No. of VFDs - Acute lung injury score - No. of days off O_2_ therapy
NCT04445220 (USA)	Target: *N* = 22	Allogenic human MSCs at 2.5 × 10^6^ cells (low dose) and 7.5 × 10^6^ cells (high dose) vs. standard care control—patients with COVID-19 and acute kidney injury	- Safety and tolerability - SAEs
NCT04276987 (China)	Target: *N* = 30	Allogenic adipose tissue derived MSC exosomes inhaled at 2 × 10^8^ nano-vesicles on 5 consecutive days	- SAEs - TTCI - No. of patients weaning from mechanical ventilation - Vasoactive agent use - No. of days on mechanical ventilation - Mortality - SOFA score - Lymphocyte count - CRP, LDH, and D-dimer - NT-proBNP - IL-1β, IL-2R, IL-6, and IL-8 - Chest imaging - Negative nCoV-test
IRCT20140528017891N8 (Iran)	Target: *N* = 10	Umbilical cord derived MSCs at 0.5–1 million cells/kg at 1st, 3rd, and 6th day vs. saline injection at 1st, 3rd, and 6th day plus standard care	- Mortality - Pneumonia severity index and CT scan - SpO_2_ supply - CRP and PCT - Lymphocyte count - T-cell analysis (CD3^+^, CD4^+^, and CD8^+^)
NCT04355728 (USA)	Target: *N* = 24	Umbilical cord derived vs. standard care control	- Adverse events - 90 day survival post-infusion - No. of VFDs - Change in oxygenation index and plat-PEEP - SOFA and SIT scores - TnI, CRP, and D-dimer - WBC and platelet count - AA/EPA ratio - 25-Hydroxyl Vitamin D - Alloantibody levels
CHICTR2000030224 (China)	Target: *N* = NA	MSCs (source unknown): critical and severe group injected with MSCs vs. critical and severe control group injected with saline	- SpO_2_ - CT scan - Temperature - Routine blood markers - Inflammatory markers - Hepatic and renal function
ChiCTR2000030173 (China)	Target: *N* = NA	Umbilical cord derived vs. standard care control	- Pulmonary function - nCoV pneumonic nucleic acid test - Pulmonary CT and chest radiography
CHICTR2000030138 (China)	Target: *N* = NA	Umbilical cord derived vs. standard care plus saline injection control	- Clinical index
ChiCTR2000030088 (China)	Target: *N* = NA	Umbilical cord Wharton's jelly derived MSCs at 1 × 10^6^ cells/kg vs. standard care and saline injection control	- nCoV pneumonic nucleic acid test - CT scan of ground glass shadow
CHICTR2000029990; TARGET *N* = NA (China)	Target: *N* = NA	MSCs (source unknown) vs. standard care and saline injection control	- Respiratory system function (O_2_ saturation) recovery time
ChiCTR2000029817 (NA)	Target: *N* = NA	Umbilical cord derived MSCs and NK cells: - High dose group: NK cells and MSCs at > 5 × 10^9^; Once every 2 days, five times - Conventional dose group: NK cells and MSCs at > 3 × 10^9^; once every 2 days, three times - Preventive dose group: NK cells and MSCs at > 3 × 10^9^; one infusion	- Time to disease recovery and time to negative nCoV test - Clearance rate and time of main symptoms - Transfer to ICU time - Routine blood tests - Biochemical indicators - Immune indices
CHICTR2000029816 (NA)	Target: *N* = NA	Umbilical cord derived MSCs (dose not stated) vs. standard care control	- Time to disease recovery and time to negative nCoV test - Clearance rate and time of main symptoms - Transfer to ICU time - Routine blood tests - Biochemical indicators - Immune indices
ChiCTR2000029580 (China)	Target: *N* = NA	Ruxolitinib and MSCs (source and dose not stated) vs. standard care control	- Safety
CHICTR2000029569 (China)	Target: *N* = NA	Umbilical cord derived blood mononuclear cells conditioned medium vs. standard care control	- PSI, CT, and X-Ray - Arterial blood gas - Assisted breathing time - Mortality - Disease evolution - Hospitalization days - Safety outcome index
EUCTR2020-001450-22-ES (Spain)	Target: *N* = NA	Allogenic umbilical cord derived MSCs (dose not stated)	- Mortality - Mechanical ventilation incidence - Need for vasopressors - Safety profile of MSCs - Neutrophils, monocytes and NK cells PCT, ferritin, D-dimer and hs-troponin - PCR test - B and T lymphocytes - Interleukins, Th1, 2&17, NLRP3, and HMGB1
IRCT20200421047150N1 (Iran)	Target: *N* = NA	Umbilical card Wharton's jelly derived: three injections at 0.5–1 million cells/kg at 1st, 3rd, and 6th day. Control receiving standard care plus saline injection at 1st, 3rd, and 6th day	- Not stated
ACTRN12620000612910 (Australia)	Target: *N* = NA	Mesenchymoangioblast derived MSCs (CYP-001) at 2 × 10^6^ cells/kg twice vs. ICU standard care control	- Not stated
NCT04361942 (Spain)	Target: *N* = 24	Allogenic MSCs (source unknown) vs. placebo (not stated)	- Withdrawal of invasive mechanical ventilation - Mortality - Patients achieving a clinical response - Patients achieving a radiological response
EUCTR2020-001266-11-ES (Spain)	Target: *N* = 100	Allogenic adipose tissue MSCs	- Efficacy and safety of administration of MSCs - Survival - Temperature - Withdrawal of mechanical ventilation - Patients transitioning to O_2_ therapy from mechanical ventilation - O_2_ therapy duration - Days in ICU - Duration of hospitalization - PaO_2_/FiO_2_ - Chest radiology - Routine blood markers - Inflammatory markers - Coagulation markers - Immune markers

*Source: https://clinicaltrials.gov/ct2/home and https://trialstreamer.robotreviewer.net/*.

*hs-CRP, high sensitivity C-reactive protein; IL-, Interleukin-; TNF-α, Tumor necrosis factor-α; SAE, Serious adverse event; HR, Heart rate; RR, Respiratory rate; PCT, Procalcitonin; VEGF, Vascular endothelial growth factor; RTRA, Return to room air; INR, International normalized ratio of blood coagulation; TEAE, treatment emergent serious adverse events; DLT, Dose limiting toxicity; VFD, Ventilator free days; BUN, Blood urea nitrogen; APACHE, Acute physiology and chronic health disease classification; AST, Aspartate aminotransferase; NEWS, National early warning score; LDH, Lactate dehydrogenase; AAT, Alanine aminotransferase; CK, Creatine kinase; TTCI, Time to clinical improvement; LIF, Leukemia inhibiting factor; PEEP, Positive end-expiratory pressure; SOFA, Sequential organ failure assessment; SIT, Small identification test; TnI, Troponin I; AA, Arachidonic acid; EPA, Eicosapentaenoic acid; nCoV, novel coronavirus; Polymerase chain reaction; NK, Natural killer; Th, T helper; NLRP3, NLR Family Pyrin Domain Containing 3; HMGB1, High mobility group box 1*.

The first clinical study undertaken in China, showed that for seven patients with COVID-19-related pneumonia, transplantation of 1 × 10^6^ MSCs/Kg/BW allogeneic MSCs was effective by restoring the balance of the immune system resulting in significant resolution of signs and symptoms of pulmonary disease (133). Before the transplantation, all patients had COVID-19-related pneumonia with symptoms of high fever, weakness, shortness of breath, and low oxygen saturation. Results showed that all symptoms had disappeared by 2–4 days after the transplantation. The oxygen saturations rose to ≥ 95% at rest, without or with oxygen treatment. This was not the case in the three placebo control patients. Among the MSC-treated patients, one severe and two mild patients were able to make a recovery and be discharged 10 days after treatment. The study found improvement was particularly dramatic for an elderly male patient in a severe critical condition (133). The improved recovery time with MSC treatment would lead to decreased hospitalization which would be vital for overwhelmed hospital wards and ICUs.

The transplanted MSCs significantly elevated IL-10 and reduced TNF-α concentrations in seven MSC transplanted patients with COVID-19-pneumonia compared to the three patients in the placebo control group receiving standard care. In the severe (*n* = 4) and critically severe (*n* = 1) patients, a significant elevation in Tregs and dendritic cells were observed after MSC administration, compared with the mild and control patients. Specifically, there was a switch from pro-inflammatory cytokine producing CXCR3^+^CD4^+^ T cells, CXCR3^+^CD8^+^ T cells, and CXCR3^+^ NK cells to CD14^+^CD11c^+^CD11b mid regulatory dendritic cell (DCreg) population, indicating improvement in immunomodulatory function. Furthermore, in the critically severe patient an over activation of T-cells and natural killer (NK) cells were evident, however, after MSC treatment, T-cells and NK cells were almost eradicated, with the CD14+CD11c+CD11b mid DCregs restored to normal levels (133). These findings demonstrate the ability of MSCs to induce their immunomodulatory benefits in a set of patients with COVID-19, restoring the balance of the immune response by attenuating the cytokine storm.

These findings have been further supported within the literature with a case study by Zhang et al. (162) demonstrating a regression of COVID-19 symptoms between 2 and 7 days post-Wharton's Jelly derived human umbilical cord MSCs administration, with a reduction in ground glass opacity and pneumonia infiltration within the lungs 6 days post-transplantation. Moreover, CD3^+^, CD4^+^, and CD8^+^ T-cells were increased and CRP, IL-6, and TNF-α concentrations were reduced. Another case report of a patient with severe COVID-19 who experienced two cytokine storms, was treated with a synergistic use of convalescent plasma and umbilical cord MSCs. Treatment resulted in lymphocyte counts returning to normal after the fourth day following convalescent plasma administration and a reduction in inflammatory markers, with a steady elevation in PaO_2_ following the administration of umbilical cord MSCs (167).

One limitation to MSC therapies for treating COVID-19 may be the expression of ACE2 and the predominant serine protease responsible for priming the SARS-CoV-2 spike glycoprotein, TMPRSS2, which may promote SARS-CoV-2 infection of transplanted cells and thus promote further spread and progression of COVID-19. However, Leng et al. (133) after performing 10x single cell RNA sequencing analysis, demonstrated transplanted MSCs are ACE2-negative and TMPRSS2-negative.

Taken together, *via* their immunomodulatory and reparative role these studies provide support to the rationale for MSC transplantation as a therapy to treat COVID-19. Moreover, whilst these studies demonstrate evidence for their use against lung damage, the suppression of pro-inflammatory markers will provide protection against damage or further damage to other organs. For example, with COVID-19 leading to myocardial injury, MSC transplantation could offer a cardioprotective role.

## MSC Transplantation Could Attenuate Damage and Facilitate Repair of the Cardiovascular System Seen With COVID-19

**Figure 1:**
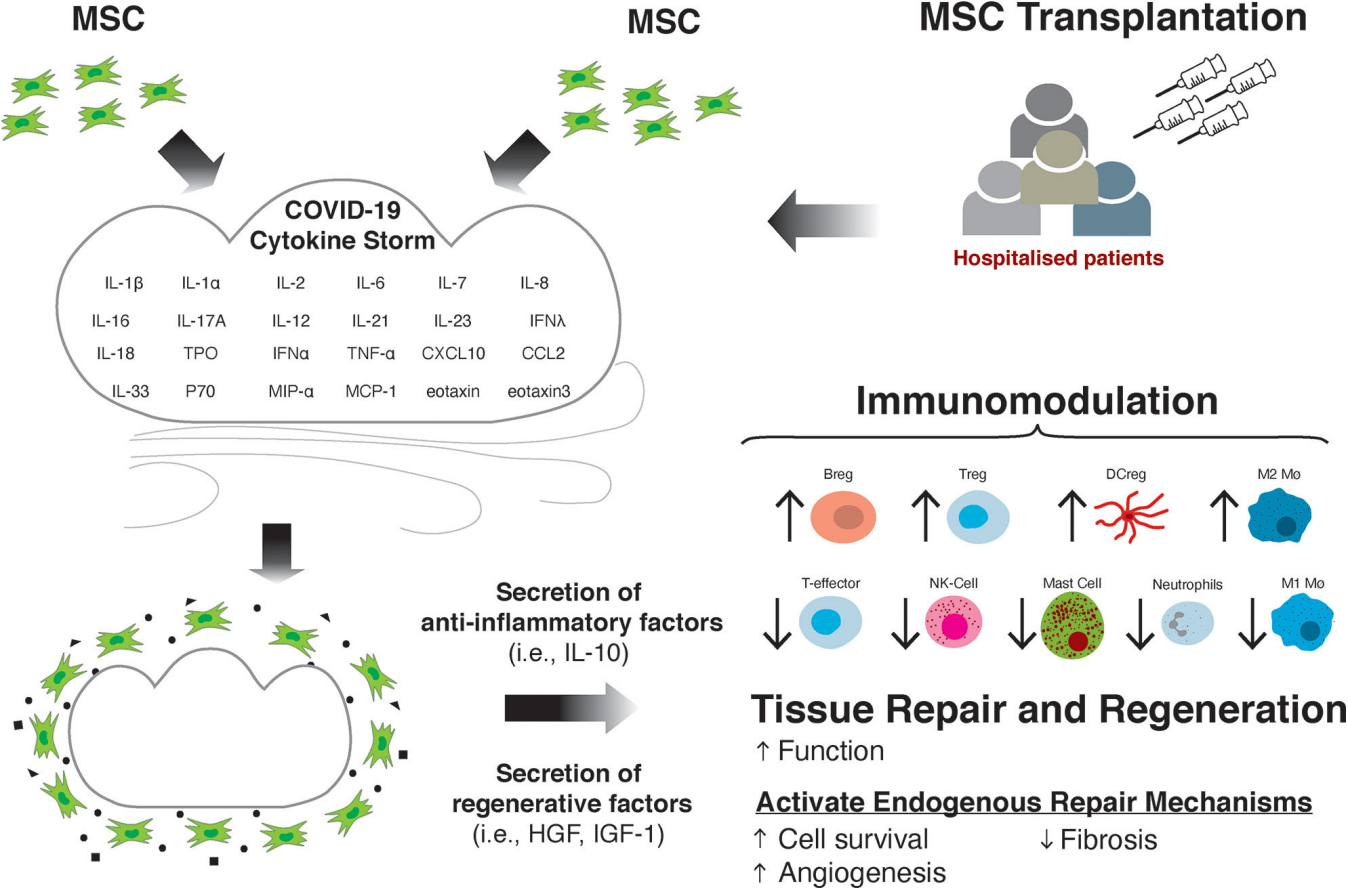
MSC transplantation attenuates the damaging effects of the cytokine storm through immunomodulation and improving tissue repair and regeneration.

In addition to the potential for MSCs to modulate the immune response and subsequent tissue damage in COVID-19, there is prospect for MSCs to treat the cardiac and cardiovascular effects of the SARS-CoV-2 virus, which may be long-lasting (Figure 1). As previously discussed, in a large proportion of patients there is evidence of myocardial injury, as suggested by elevated cTnI and cTnT levels (16, 19, 168, 169), and ventricular dysfunction indicated by raised circulating NT-proBNP (29, 31). Elevated cardiac biomarkers are associated with more severe prognosis and mortality in COVID-19 patients (18, 26, 29, 169, 170), suggesting the cardiac effects of the virus can drive worsening prognosis for the patient. Moreover, there are a number of studies detailing the severe cardiac effects of the virus, such as the development of heart failure (HF) (28), as well as incidences of acute coronary syndromes (ACS) (171, 172), ischaemic stroke (173) and myocardial infarction (MI) (171, 172). Given the significant deleterious effect of the virus on the myocardium, treatment options to minimize or to alleviate the cardiovascular side effects of the infection and disease are needed.

Treatment with MSCs may offer a clinical benefit to patients due to their regenerative and reparative potential if there is significant myocardial injury and myocardial cell death. There have been a number of studies investigating the use of autologous (174–180) or allogeneic MSCs (178, 181–184) for the treatment of cardiomyopathies and post-MI. Although the use of MSCs to treat cardiovascular dysfunction and damage in COVID-19 patients has yet to be fully elucidated, the studies over the past decade provide good preliminary evidence for researchers and clinicians alike to further investigate the use of this cellular therapy in COVID-19 patient cohorts.

Several studies in pig, rat and mouse models of MI showed significant reduction in infarct size or fibrosis (185–194), and improvements in cardiac function (185–187, 189, 190, 195, 196). A meta-analysis of 52 pre-clinical animal studies of cell therapy for ischaemic heart disease reported that MSC therapy is safe and associated with significant ~7.5% improvements in LVEF (197). In order to elicit increased efficacy, cell combination therapy has been investigated. In swine models of MI, human bone marrow-derived MSCs and cardiac-derived stromal MSC stem/progenitor cells from autologous or allogeneic sources were co-injected into the border zone of the infarct. Results showed that by combining the cell types there was greater therapeutic efficacy, improving cardiac repair/regeneration and LV functional recovery without adverse immunologic reaction (198, 199).

These promising findings have been followed by a number of human clinical trials. In a number of these human studies, the infusion and transplantation of MSCs have been deemed safe for treating MI patients (179, 200) as well as having been successful in improving some cardiac functional measures post-MI, such as LVEF (175, 177, 200–204), and improving global longitudinal strain measures (201). Penn et al. (204) showed in a phase I clinical trial in patients with first ST-elevation–myocardial infarction (STEMI), delivery of MSCs (MultiStem) using a coronary adventitial delivery system was well-tolerated and safe. In patients who exhibited significant myocardial damage, the delivery of ≥50 million MultiStem resulted in improved EF and stroke volume 4 months later (204). However, some of these studies, and others, found no difference between MSC treatment and no treatment/placebo on infarct size or perfusion changes in the months following the enrolment to the study (177, 205, 206). Additionally, several human studies fail to observe any clinical benefit for patients (179, 184, 205, 207). Inconsistent findings are likely due to the number and phenotype of MSCs being transplanted, their source, as well as mode and location of administration (myocardial, epicardial, or endocardial injection; systemic transplantation).

Despite mixed findings on the efficacy for improving cardiac function, MSCs can offer potential as regenerative cells for the CV system, where through a paracrine mechanism they activate endogenous repair mechanisms leading to blood vessel growth *via* angiogenesis, improved cardiomyocyte survival, reduced cardiomyocyte reactive hypertrophy, and fibrosis (Figure 1). We have clonally derived (from a single cell) a population of stromal cells with multipotent stem/progenitor cell properties from the adult mammalian heart, including human (208–210). These cells produce a repertoire of pro-survival and cardiovascular regenerative growth factors. We administered these cells intracoronary at differential doses (5 × 10^6^, 5 × 10^7^, and 1 × 10^8^) in three groups of white Yorkshire female pigs with MI, 30 min after coronary reperfusion. Pig serum was injected to six control pigs after MI. We found a high degree of cell engraftment in the damaged pig myocardium. By 3 weeks after MI and cell transplantation, there was increased new cardiomyocyte and capillary formation, which was not evident in the control hearts (194). Moreover, cell treatment preserved myocardial wall structure and attenuated remodeling by reducing cardiomyocyte hypertrophy, apoptosis, and scar formation (fibrosis) (211).

In mouse, rat and *in vitro* cell model studies, MSCs have been found to be potently angiogenic (192, 212–221). As outlined previously, MSCs most likely promote angiogenesis *via* paracrine means, such as secretion of angiogenic factors; vascular endothelial growth factor (VEGF), basic fibroblast growth factor (bFGF), transforming growth factor beta (TGF-β), and platelet-derived growth factor (PDGF) (222, 223), which are promoted under hypoxic conditions (224). Proteomic analysis of secreted exosomes, which carry lipids, proteins and genetic material to target tissues, from MSCs reveal several target pathways (225). These include inflammation and angiogenesis, of which, the angiogenesis pathway revealed specific interaction with NF-κ-B signaling. When these exosomes were cultured with HUVECs, a significant increase in endothelial tube formation was detected in a dose-dependent fashion (225). Zhang et al. (226) investigated the potential for MSC-derived exosomes to promote angiogenesis and cardiac repair post-MI in rats. Firstly, they observed that exosomes isolated from MSCs promoted tube formation of cardiac stem/progenitor cells *in vitro*. They subsequently transplanted cardiac stem/progenitor cells internalized with these exosomes into a rat model of MI, and observed an increased capillary density, which was followed by an improvement in LVEF, and reduction in fibrosis after 28 days post-implantation. Interestingly, the source of MSCs can significantly alter their pro-angiogenic potential. Du et al. (219) isolated MSCs from bone marrow, adipose tissue, umbilical cord and placenta and assessed their pro-angiogenic capacity using *in vitro* tube formation assays, as well as endothelial cell proliferation and assessment of angiogenic gene expression by RT-PCR. They found that MSCs isolated from the bone marrow and the placenta promoted angiogenesis *in vitro* to a greater extent than MSCs from adipose tissue and umbilical cord. In addition, they found that MSCs from these sources had a greater expression of VEGF mRNA and protein (219).

As well as promoting angiogenesis, MSCs may promote recovery from cardiac injury/insult by differentiating into mature cardiomyocytes, or by promoting resident cardiomyocyte proliferation. Mesenchymal stromal cells have a broad differentiation capacity, and have been shown to be able to differentiate into osteoblasts (227), neuronal cells (228) as well as upregulate cardiomyocyte markers, such as cardiac myosin heavy chain (229) and troponin T (229, 230). However, several studies have failed to observe significant trans-differentiation of MSCs into either endothelial cells or functional cardiomyocytes (189, 231, 232). Otherwise, MSCs have been found to promote cardiomyocyte DNA synthesis and proliferation, and signal cardiomyocyte gene upregulation (including VEGF, cyclin A2, and TGF-β2) (194, 233). Through their paracrine activity, they also prevent cardiomyocyte cell apoptosis (188, 221, 234–236) with several studies observing a reduced activation of the caspase-3 pathway in cardiomyocytes exposed to either MSC-derived exosomes (236) or conditioned media (237).

Other methods to maximize cellular function of cell therapies include “priming” which involves promoting expression of certain receptors, proteins and cytokines in the cells prior to transplantation or infusion. Mesenchymal stromal cells primed *in vitro*, prior to *in vivo* administration may offer opportunity to improve the efficacy of MSC treatment. Several studies have shown that by priming these cells *in vitro*, for example to highly express GATA-4 (MSC^GATA−4^) (238), or CXCR4 (MSC^CXCR4^) (233, 239) may improve the angiogenic paracrine activity of these cells. Mesenchymal stromal cells which were overexpressing GATA-4 contained more VEGF and IGF-1 protein, which, when blocked with neutralizing antibodies, attenuated the pro-angiogenic activity of MSC^GATA−4^ (238). Moreover, cardiac-derived stem/progenitor cells that express high levels of GATA-4 have shown to foster cardiomyocyte survival through IGF-1 paracrine signaling (240). MSC^CXCR4^ cells themselves were found to be highly angiogenic compared to un-primed MSCs, with greater expression of VEGF, which may partly explain the greater *in vitro* tube formation observed in a study by Zhang et al. (239). CXCR4 over-expression may be beneficial in promoting cell migration to ischaemic tissue due to the ligand stromal-derived factor-1 (SDF-1) (241), which is released in ischaemic tissue (242, 243). Thus, by selecting CXCR4^+^ MSCs, or promoting CXCR4 expression *in vitro*, MSC migration to target infarct or damaged areas may be improved, subsequently allowing the cells to stimulate repair in the area required more efficiently.

Heart tissue damage post-MI, although largely due to ischaemic tissue injury and insult and associated cardiomyocyte loss, is also due to inflammation associated in the hours and days post-MI (244, 245). This inflammatory response is associated with further cardiac tissue damage and injury, as indicated by sustained and continual increases in cTnI and cTnT (246). Indeed MSC exosomes can regulate T-cell proliferation (215) as well as alter the balance between M1 and M2 macrophages in the infarcted heart (191), and the number of neutrophils and NK cells post-MI in the cardiac tissue (244) suggesting strong anti-inflammatory properties of the MSCs. In fact, a study by Luger et al. (244) found that MSC exosomes were able to reduce the number of NK cells in cardiac tissue post-MI, followed by a separate experiment whereby depleting NK cells 24 h prior to MI in mice, reduced the resulting infarct size. These findings infer that NK cells are involved in causing, or significantly contributing to, the cardiac damage resulting from an ischaemic challenge, and that MSCs could attenuate this inflammation. Taken together, it appears that MSCs also promote cardiac recovery *via* attenuating the ongoing inflammatory response, which is also a likely pathway for COVID-19-associated myocardial injury.

Although there is significant promise in the use of MSCs for cellular therapy to treat cardiovascular conditions, their efficacy for use in treating COVID-19-related cardiac dysfunction and injury is yet to be determined.

## MSC Transplantation in COVID-19 Patients Could Alleviate Pulmonary Fibrosis

Fibrotic disorders in the lung, such as idiopathic pulmonary fibrosis (IPF), share similar comorbidities with COVID-19. Both conditions are progressive in nature, often because of worsening lung injury and fibrosis of alveolar walls. This underscores a common anti-fibrotic strategy.

Clinical trials with anti-fibrotic agents have shown promise in reversing progression of pulmonary fibrosis, as evidenced with nintedanib (247) and pirfenidone (248), which were approved by the FDA more than 6 years ago (249). This is supported by findings from pre-clinical animal models. An animal model of IPF with increased fibrosis and defective clearance of fibrocytes and myofibroblasts, was improved upon treatment with nintedanib (250). However, whether these agents will have clinical efficacy in COVID-19 remains unknown. Notably, commercial anti-fibrotic drugs, such as nintedanib and pirfenidone, are only available for oral delivery. This limits their use in COVID-19 patients, given that the population with fibrotic lung damage are usually hospitalized and intubated. Moreover, the hepatoxic side effects of both drugs and the contraindication of pirfenidone in renal dysfunction further limit their use, especially noting that SARS-CoV-2 is associated with development of both liver and kidney dysfunctions (58, 251). This highlights the need for better therapeutic strategies for lung fibrosis. Novel treatment options, such as cell-based therapy for replenishing lost functional capacity of resident stromal cells, have great potential for patients with COVID-19.

Cell-based therapy has been keenly investigated in the pre-clinical models using bleomycin-induced pulmonary fibrosis. Bleomycin-induced lung injury is a well-characterized model of human pulmonary fibrosis, with an initial phase of inflammatory activation and consequent fibrosis. In mice, intravenous injection of the primary human amniotic epithelial cells (hAECs) reduced lung inflammation and expression of the pro-fibrotic ligand TGF-β1 (252). Human amniotic epithelial cells transplantation also reduced the Ashcroft score, a validated marker of severity of lung fibrosis (253), likely due to increased degradation by matrix metalloproteinase (MMP)-2 and reduced expressions of tissue inhibitors of MMPs (TIMP)-1 and 2 (252). A pooled analysis of pre-clinical evidence demonstrated significantly better results on Ashcroft score and collagen contents for hAECs compared to placebo (254). Much akin to hAECs, MSCs have been shown to ameliorate pulmonary injury induced by bleomycin in experimental models (255). This has been demonstrated for bone marrow, umbilical cord, and amniotic fluid derived MSCs, respectively. The therapeutic efficacy of MSCs is also reported in other models of lung fibrosis. For example, adipose tissue-derived MSCs significantly attenuated lung function and fibrosis in a rodent model of silica-induced lung fibrosis (256). In summary, these data show that MSC-based therapy is a promising tool to address the pathophysiological consequences of COVID-19 in the lung. However, clinical translation would require more refined understanding of the anti-fibrotic mechanisms of MSCs.

Cumulative data show that MSCs protect against fibrosis *via* hepatocyte growth factor (HGF)-mediated mechanisms. Hepatocyte growth factor was originally identified as a mitogen for hepatocytes. It has now been shown to mediate mitogenic, anti-inflammatory, anti-apoptotic, and regenerative effects during tissue repair. In models of I/R lung injury, transplanted HGF-overexpressed MSCs resulted in lessened oxidative stress, inflammation, and attenuated lung injury (257). Hepatocyte growth factor also prolonged the survival of engrafted MSCs *via* increased expression of the anti-apoptotic protein Bcl-2 and repression of caspase-3 activation. In the context of fibrosis, there is evidence to suggest that HGF modulates pro-fibrotic pathways. For instance, microvesicles from human Wharton's Jelly MSCs inhibited apoptosis, fibrosis in pulmonary tissues, and activation of PI3K/AKT/mTOR pathway (258). These effects were blocked by using HGF-mRNA-deficient microvesicles or PI3K inhibitor. Hepatocyte growth factor also inhibits alveolar epithelial-to-mesenchymal transition and production of TGF-β1 independent of MSCs (259).

Other pathways have also been implicated in mediating the anti-fibrotic role of MSCs, including the activation of MMP-9 (260), programmed death (PD)-1/PD-L1 (261), and anti-apoptotic Bcl-2 (256, 257). MMP-9 is said to promote the degradation of collagen deposits, thereby facilitating the repair process following lung injury. On the other hand, MSC transplantation has been associated with repressed TGF-β1/SMAD3 (255), Wnt/β-catenin signaling (262), MyD88/TGF-β1 signaling (263), and N-methyl-d-aspartate receptor activity (264). Inhibition of Wnt/β-catenin signaling has a two-fold function. Firstly, it prevents downstream activation of pro-fibrotic genes and development of fibrosis; and, secondly, it rescues lung resident MSCs from differentiating to myofibroblasts (265).

Whether similar benefits will be seen in COVID-19 patients remains to be established. A single center, non-randomized, dose-escalation phase 1b trial of eight patients with moderate-to-severe IPF treated with intravenous bone marrow-derived MSC showed a good short-term safety profile (266). CT fibrosis score did not change 6 months after administration compared to baseline; however, there was no further worsening of fibrosis during follow-up. Similar findings were noted in a larger (randomized) trial of 20 IPF patients treated with high-dose bone marrow-derived MSCs (267). Subsequently, a trial of 61 patients with influenza A (H7N9)-induced ARDS showed significant reduction in the inflammatory marker CRP following menstrual-blood-derived MSC treatment, compared to placebo (163). While treated patients showed linear fibrosis, ground-glass opacity, and pleural thickening on chest CT at baseline, there was improvement in all patients after 24 weeks and up to 1 year after MSC treatment.

Our current understanding of the mechanisms of MSC-mediated improvement in lung (fibrotic) injury is incomplete, especially in the context of COVID-19. There are other important questions that will need to be addressed, too. For instance, would the MSCs need to be primed for improved efficacy? Previous studies have shown that pre-conditioning of MSCs with oncostatin M (268, 269), low-dose TGF-β1 (270), IL-6 (269), or ischaemia (271) improves the survival and therapeutic benefits. Obtaining the best MSCs for transplantation in terms of optimum immunomodulatory capacity and availability should be considered in COVID-19 studies. Primary MSCs, such as those obtained from bone marrow, umbilical cord, or adipose tissue, are limited by lack of available donors, many lack standardized preparations, with variations in quality, limited regenerative capacity, and finite lifespans. To overcome these limitations, a recent study investigated a novel hESC-derived MSC-like cell population, termed Immunity-and Matrix-Regulatory Cells (IMRCs) (272). Produced to good manufacturing standards, IMRCs demonstrated excellent safety and efficacy profiles in *in vivo* models of mice and monkeys. Additionally, IMRCs demonstrated superior immunomodulatory effects compared to umbilical cord-derived MSCs and the anti-fibrotic agent, pirfenidone (272).

## Conclusion

Evidence now supports severe COVID-19 being associated with a dysregulated and hyperactive inflammatory systemic response; a cytokine storm. Older people (>60 years) and people with co-morbidities are more likely to develop a dysfunctional immune response, and resultant cytokine storm, that causes pathology and fails to successfully eradicate the pathogen. The exact reasons for this are unclear, although one reason may be a decline in immune function with age and chronic sterile inflammation due to the build-up of senescent cells and immunosenescence in aging humans (273).

The manifestations of elevated pro-inflammatory, sustained circulating factors due to the cytokine storm are not just confined to the lungs, with significant damage to the CV system and multi-organ damage and dysfunction. Interventions that target single cytokines (i.e., Tocilizumab targeting IL-6) do not seem efficacious in reducing mortality. Mesenchymal stromal cells owing to their powerful immunomodulatory function can holistically target and suppress the cytokine storm. At the same time, MSC transplantation is safe and has proven effective at activating endogenous repair mechanisms, leading to improved cardiac function, tissue regeneration and decreased fibrosis. Therefore, attenuating persistent organ dysfunction. Further mechanistic studies are required to investigate if MSC therapy can alleviate the cardiovascular consequences of COVID-19, and thus reduce cardiovascular risk in these patients. Work should also focus on determining the optimal dose, timing of injections (multiple dosing at different stages of the disease), systemic distribution of transplanted cells, type of MSCs used or use of exosomes, and the anti-viral effects of MSC transplantation.

## Author Contributions

LC put together the tables. TA put together the figure. GE-H oversaw the completion of the article. All authors contributed to writing the article.

## Conflict of Interest

The authors declare that the research was conducted in the absence of any commercial or financial relationships that could be construed as a potential conflict of interest.
